# Oral mucosa graft ureteroplasty: defining its role in complex ureteral reconstruction

**DOI:** 10.1097/MOU.0000000000001394

**Published:** 2026-04-02

**Authors:** Mattia Lo Re, Bruno Bucca, Andrea Cocci

**Affiliations:** a Department of Experimental and Clinical Medicine, University of Florence, Florence, Italy; b Unit of Urology and Andrology, Careggi Hospital, Florence; c Department of Urology “U. Bracci”, Policlinico Umberto I Hospital, Sapienza University of Rome, Rome, Italy

**Keywords:** buccal mucosa, oral mucosa, ureteral obstruction, ureteral stricture, ureteroplasty

## Abstract

**Purpose of review:**

Oral mucosa graft ureteroplasty has emerged as an organ-sparing option for complex ureteral strictures, potentially avoiding bowel interposition or renal autotransplantation in selected patients. This review is timely because indications, technical variants, and outcome definitions remain heterogeneous, and contemporary series are expanding the evidence base.

**Recent findings:**

Across predominantly retrospective cohorts, oral mucosa grafting is most frequently applied to proximal and mid-ureteral strictures of several centimetres, including redo and post-endoscopic etiologies. Reported success is high when a well-vascularized bed is ensured and postoperative surveillance is standardized. Technical themes include onlay versus augmented anastomotic repairs, the selective use of vascularized coverage (omental or retroperitoneal/perinephric fat), and growing attention to donor-site morbidity, which is generally low but inconsistently reported.

**Summary:**

Oral mucosa graft ureteroplasty should be considered within the reconstructive ladder for carefully selected complex strictures where primary anastomosis or reimplantation is not feasible, and where more morbid substitutes can be deferred. Future priorities include standardized definitions of success, comparative studies against ileal ureter and autotransplantation, and long-term renal functional outcomes with patient-reported measures.

## INTRODUCTION

Complex ureteral strictures and long-segment ureteral defects remain a persistent source of renal deterioration and repeated procedures, most commonly following stone-related interventions, iatrogenic injury, trauma, radiation, or benign inflammatory disease [1^▪^].

The most common cause of iatrogenic injury is laparoscopic gynaecologic surgery (64%), which often involves the distal third of the ureter [2].

General surgical procedures (26%) and urologic procedures (11%) are also associated with iatrogenic ureteral injury [1^▪^,^3^].

The classic reconstructive ladder, from primary ureteroureterostomy or ureteroneocystostomy with adjunct bladder mobilization to bowel substitution or renal autotransplantation, can restore drainage but may be limited by defect length, compromised vascularity, and the substantial morbidity of bowel interposition or transplant surgery in selected patients [2,4^▪^]. In fact, bowel interposition remains a dependable option for extensive ureteral loss with defects from 8 to 10 cm or pan-ureteral disease. Contemporary series report success rates of 83–97%, but with complications that include small bowel obstruction, anastomotic fistula, incisional hernia, and hyperchloremic metabolic acidosis [2,5]. For selected right-sided proximal or mid ureteral strictures, appendiceal ureteroplasty can be a tissue-sparing alternative. Robotic series report success rates of 81–100% for strictures around 2.5–6.5 cm [5]. Practical limitations include appendiceal length variability, prior appendectomy, right-sided anatomy, and the need for a transperitoneal approach [5]. In this setting, buccal mucosa graft (BMG) ureteroplasty, first described by Naudé in 1999 [6], has emerged as an appealing organ-preserving strategy that augments the ureteral plate while maintaining native tissue continuity. Current European Association of Urology guidance on complex ureteral injury acknowledges BMG ureteroplasty as a viable option when conventional repairs are not feasible or have failed, while recognizing that the evidence base continues to evolve [3]. The last contemporary systematic review included 16 studies and 398 patients, reporting consistently high success rates across surgical approaches, exceeding 85% in all studies [7^▪^].

This review therefore aims to define where BMG ureteroplasty fits within contemporary practice by synthesizing indications, outlining the principal technical variants and coverage strategies, and summarizing reported outcomes, including donor-site morbidity and renal function preservation to support clearer decision-making and identify priorities for future investigation. 

**Box 1:**
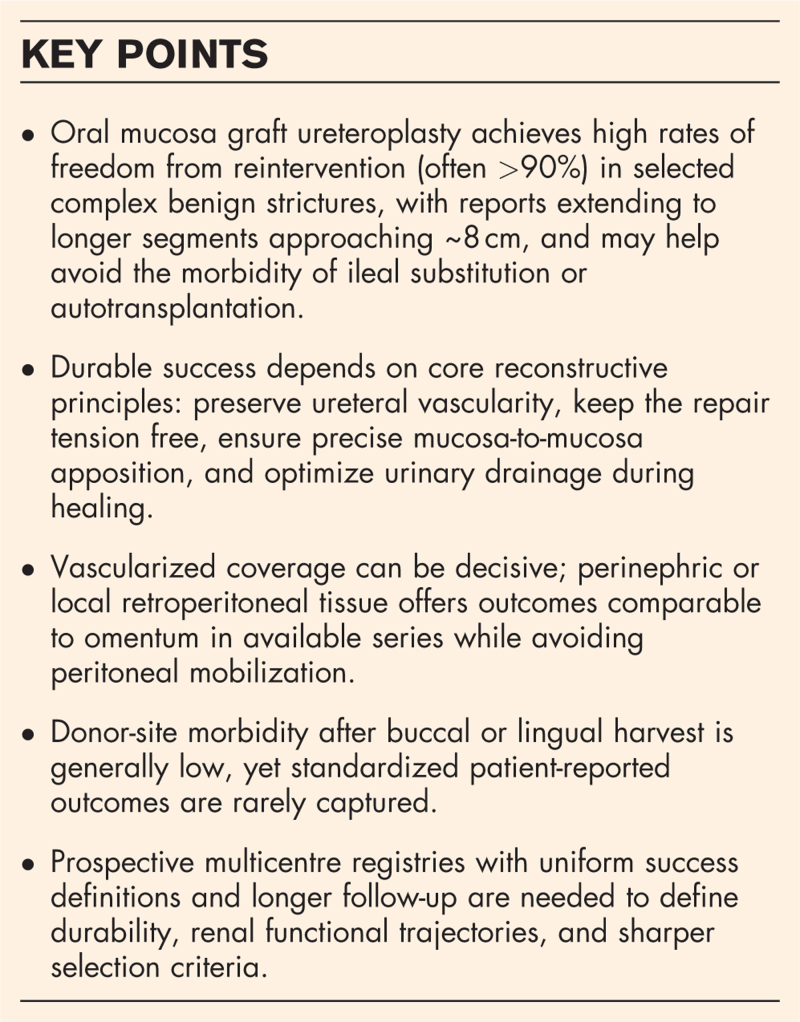
no caption available

## CLINICAL CONTEXT AND INDICATIONS

Complex ureteral strictures encompass excessive length (typically >3 cm), unfavourable proximal or mid-ureteral location, dense periureteral fibrosis from prior intervention, or compromised tissue vascularity from radiation or ischemia [8]. Understanding the underlying etiology is essential for surgical planning and prognostication.

### Etiologies and location-specific considerations

As reported in the introduction, iatrogenic strictures are the most common. Among them, radiation-induced strictures present challenges due to microvascular compromise extending beyond visible disease margins [9^▪▪^]. Endometriosis affects approximately 1% of women with the disease, predominantly involving the distal ureter, with 26% diagnosed only at final pathology – highlighting the importance of high clinical suspicion in premenopausal women [10^▪^]. Depending on the ureteral segment affected, the surgical indication changes.

The ureter is classically divided into proximal, mid, and distal segments. The proximal ureter extends from the ureteropelvic junction to the sacroiliac joint, the mid ureter traverses the pelvis, and the distal ureter runs from the iliac vessels to the bladder. Blood supply derives from the renal artery for the proximal segment, the common iliac artery for the mid ureter, and the superior vesical artery for the distal ureter.

Ureteroureterostomy is a successful technique for short (<3 cm) strictures of the middle and distal ureter [8].

Anatomically, distal strictures remain amenable to ureteroneocystostomy with psoas hitch or Boari flap, whereas proximal and mid-ureteral defects exceeding 2–3 cm often preclude tension-free primary anastomosis, creating the ideal scenario for oral mucosa graft augmentation [2,9^▪▪^,11^▪^].

### Patient selection and stricture length limits

Patient selection critically influences outcomes. In the largest multi-institutional series (163 patients), Chao et al. included strictures with median length 3.0 cm (IQR 2.0–4.0), predominantly proximal (55%) and mid-ureteral (34%), with 47% having failed prior treatment [9^▪▪^]. In the last meta-analysis published, Zeng *et al.*'s data demonstrate successful repair of strictures up to 8 cm using both buccal and lingual mucosa grafts [11^▪^]. Contraindications for BMG augmentation include active oral mucosal disease, prior head/neck radiation compromising graft quality, while in cases of severe atrophic kidneys (<15–20% split function), nephrectomy may be preferable [12,13^▪^]. Radiation-induced strictures require meticulous assessment of tissue viability, with indocyanine green fluorescence emerging as a valuable intraoperative adjunct [9^▪▪^,14^▪^].

## PREOPERATIVE ASSESSMENT AND PLANNING

Thorough preoperative evaluation is paramount to ensure appropriate patient selection and optimize surgical outcomes. A multimodal imaging approach enables precise delineation of stricture characteristics and assessment of renal function.

### Imaging and functional work-up

Cross-sectional imaging with computed tomography urography (CTU) constitutes the cornerstone of anatomical assessment, providing detailed information on stricture location, length, and relationship to adjacent structures [2]. Retrograde or antegrade pyelography remains valuable for precise stricture characterization when cross-sectional imaging is inconclusive, particularly in delineating the proximal and distal extent of disease and in cases with a fixed percutaneous nephrostomy [9^▪▪^]. Nuclear renography with mercaptoacetyltriglycine (MAG3) or diethylenetriaminepentaacetic acid (DTPA) is mandatory in cases of suspected hypofunctional kidney to confirm obstruction, quantify split renal function, and guide decision-making regarding reconstruction versus nephrectomy; kidneys contributing less than 15–20% of total function may be better served by radical surgery [13^▪^].

### Drainage strategy and timing

In symptomatic patients, urinary diversion with percutaneous nephrostomy or ureteral stenting is recommended, allowing resolution of active infection, flank pain, optimization of renal function, and reduction of periureteral inflammation [14^▪^]. Some authors also described that positioning a ureteral stent for 6–12 weeks before surgery could play a role as a protective factor for relapse-free surgery; in Lee et al., patients undergoing ureteral rest were associated with a higher success rate compared to those not undergoing ureteral rest (90.7% versus 77.5%, respectively; *P* = 0.027) [15].

## SURGICAL PRINCIPLES OF ORAL MUCOSA GRAFT URETEROPLASTY

The fundamental tenets of successful ureteroplasty closely parallel those established in urethral reconstruction: preservation of native blood supply, creation of a well-vascularized graft bed, tension-free reconstruction, and reliable urinary drainage during healing [2,16^▪^]. Key point is not the graft itself, but the quality of the recipient bed and how much ureter must be mobilized. For these reasons, the operative strategy should be built around two decisions: whether a viable ureteral plate remains, and whether vascularized coverage is needed to support graft take.

### Graft harvest

Both buccal and lingual mucosa provide excellent donor tissue, characterized by a thick, non-keratinized epithelium, dense capillary network, and thin lamina propria that facilitates rapid neovascularization [6,11^▪^]. Buccal mucosa is harvested from the inner cheek, typically yielding grafts of 4–6 cm in length and 1.5–2 cm in width; the parotid duct orifice (Stensen's duct) should be identified and preserved to reduce the risk of salivary complications [4^▪^,14^▪^]. Lingual mucosa, obtained from the ventrolateral tongue surface, offers comparable histological properties and is a practical alternative when buccal tissue is unavailable or insufficient. Meta-analysis suggests slightly higher success rates (99% versus 95%) and lower donor site morbidity with lingual grafts, although direct comparative trials are lacking, and selection effects cannot be excluded [11^▪^]. After harvest, the graft is defatted and trimmed to match the ureteral defect length, then kept moist in saline solution until implantation.

### Ureteral preparation and graft placement

Adequate ureteral mobilization is required to achieve a tension-free reconstruction while preserving the longitudinal blood supply within the adventitia. The stricture segment is identified, when available, aided by intraoperative retrograde injection of methylene blue or indocyanine green [14^▪^], and the ureter is opened longitudinally through the entire stenosis into healthy proximal and distal tissue The choice between ventral (anterior) and dorsal (posterior) onlay positioning should follow exposure and tissue quality. Dorsal placement can exploit the vascularized retroperitoneal bed, whereas ventral onlay can be more straightforward to execute, particularly in robotic settings [17,18]. The graft is secured with running or interrupted 4 0 or 5 0 absorbable monofilament sutures (poliglecaprone or polydioxanone), ensuring tension-free mucosa to mucosa apposition [4^▪^,19^▪^,^20^].

### Technique variants

The onlay technique is preferred when a preserved posterior ureteral plate is present; the ureter is incised longitudinally and the graft augments the lumen without segmental excision [4^▪^,19^▪^]. Augmented anastomotic ureteroplasty is better suited for obliterative strictures, where simple augmentation is not feasible; the diseased segment is excised, posterior walls are approximated with interrupted sutures, and the graft closes the remaining anterior defect that would otherwise be under tension [9^▪▪^,^20^]. When the posterior wall remains inadequate even after partial approximation, Chen and colleagues described a combined posterior inlay with an anterior onlay configuration to address near circumferential defects [21]. More recently, a non-transecting mucosa-to-mucosa anastomosis approach has been reported, adapting the same non-transecting principles from urethroplasty with the specific intent of preserving segmental ureteral vascularity while restoring the lumen through mucosal approximation followed by graft augmentation [16^▪^].

### Vascularized coverage strategies

Ensuring adequate blood supply to the graft is a recurring determinant of success across series. Omental wrapping has traditionally been used as the default strategy, with the flap mobilized on its gastroepiploic pedicle, passed through a window in the transverse mesocolon when needed, and secured around the graft with absorbable sutures [4^▪^,^9^^▪▪^]. Zhou and colleagues described a simplified approach in which the omentum is draped over the graft rather than threaded dorsally, reducing technical complexity while maintaining similar reported outcomes [22,23]. Perinephric fat wrapping, as described by Jiang and colleagues, achieved equivalent success to omental coverage in laparoscopic reconstruction of proximal ureteral strictures and was associated with faster bowel recovery (1.0 versus 1.9 days, *P* = 0.009) [24^▪^]. Engelmann and colleagues further questioned the necessity of formal wrapping, reporting 93% success in 14 patients with coverage by native retroperitoneal fat alone [12].

### Stenting and drainage

A double J stent is typically placed to maintain luminal patency and divert urine flow during graft healing, with removal typically performed at 4–8 weeks after confirmatory imaging [9^▪▪^,19^▪^]. Retroperitoneal drainage is often used for 24–48 hours to detect early urinary extravasation. In particularly complex reconstructions, some authors advocate adjunct nephrostomy drainage to maximize diversion during the early healing phase, especially when tissue quality is uncertain or prior failures raise the cost of a leak [4^▪^].

Summary of techniques and outcomes in Tables 1 and 2.

**Table 1:** Surgical technique variants and vascularized coverage strategies in oral mucosa graft ureteroplasty

Technique	Indication	Key technical points	Advantages	Limitations
Onlay (ventral)	Patent lumen, anterior stricture	Longitudinal ureterotomy, graft sutured to ureteral edges anteriorly	Easier robotic access, good visualization	Less vascularized bed anteriorly
Onlay (dorsal)	Patent lumen, posterior access feasible	Ureter rotated, graft placed on retroperitoneal tissue bed	Vascularized tissue support posteriorly	Technically demanding exposure
Augmented anastomotic	Obliterative stricture, complete occlusion	Excision of diseased segment, posterior anastomosis, anterior graft coverage	Complete removal of diseased tissue	Requires adequate ureteral length
Posterior-inlay + anterior-onlay	Near-circumferential defect	Combined configuration for extensive defects	Addresses extensive circumferential defects	Complex technique, limited published data
Non-transecting	Narrow ureteral plate, redo cases	Mucosal approximation without transection, followed by graft augmentation	Preserves segmental ureteral vascularity	Early experience, validation needed
Omental wrap	Standard, transperitoneal approach	Pedicled omental flap secured circumferentially around graft	Robust vascularization, lymphatic drainage	Requires peritoneal entry, bowel manipulation
Perinephric fat wrap	Retroperitoneal approach	Local perirenal adipose tissue secured over graft	Faster bowel recovery, no peritoneal entry	Limited tissue in thin patients
No formal wrap	Selected cases, healthy non-irradiated tissue	Graft covered by native retroperitoneal fat only	Simplified procedure, reduced operative time	Requires optimal tissue quality, careful selection

AAU, augmented anastomotic ureteroplasty; MsANTA, mucosal-sparing augmented non-transected anastomotic.

**Table 2:** Published series on oral mucosa graft ureteroplasty: surgical approach, technique, and outcomes

Author, year	*n*	Approach	Graft	Stricture length (cm)	Coverage strategy	Success (%)	Follow-up (months)
Chao *et al.*, 2025	163	Robotic	BMG	3.0 (IQR 2.0-4.0)	Omental (majority)	92.0	29 (median)
Zhao *et al.*, 2018	69	Robotic	BMG	3.0 (mean)	Omental	92.7	26 (median)
Sahay *et al.*, 2024	16	Lap/Robotic	BMG	5.28 (mean)	Omental/fat	93.75	12 (mean)
Nasef *et al.*, 2025	21	Open	BMG	4.1 (mean)	Omental	85.7	16.3 (mean)
Jiang *et al.*, 2024	26	Laparoscopic	OMG	2.3-3.6	Omental versus PFW	Equivalent	36.8 (mean)
Engelmann *et al.*, 2024	14	Robotic	BMG	1.7 (median)	None	93.0	15 (median)
Yang *et al.*, 2022	32	Robotic	LMG	4.2 (mean)	Omental	96.9	24 (median)
Zeng *et al.*, 2025 (meta)	436	Mixed	BMG/LMG	Up to 8	Various	95-99	Variable

BMG, buccal mucosa graft; LMG, lingual mucosa graft; OMG, oral mucosa graft; PFW, perinephric fat wrap; Lap, laparoscopic.

### Open, laparoscopic and robotic approach

For many years, the open approach was regarded as the gold standard, achieving reported success rates between 87.5% and 94.1%, as documented in a 2021 systematic review [25]. However, technological advancements and the widespread adoption of minimally invasive techniques have progressively reshaped the surgical management of numerous urological conditions. The first laparoscopic application of buccal mucosa graft ureteroplasty was described by Li *et al.* in 2015, who reported a successful outcome in a single patient with proximal ureteral stricture [26].

In the only comparative study published, a retrospective multicentre series, both open and robot-assisted ureteroplasty using BMG demonstrated high success rates and acceptable perioperative morbidity, with no significant differences in overall postoperative complication rates between the two approaches [27]. The robotic approach was associated with significantly lower intraoperative blood loss compared with open surgery (median 175 ml versus 300 ml; *P* = 0.03), while maintaining comparable functional success rates (approximately 93.7% for robotic versus 90% for open repair). No major intraoperative complications were reported in either group. Overall, these findings suggest that robot-assisted BMG ureteroplasty is at least non-inferior to the open approach in terms of functional outcomes, while potentially offering perioperative advantages such as reduced blood loss and lower surgical morbidity.

In the most recent systematic review, Ditonno *et al.* reported that pooled success rates were comparable across approaches, reaching 90.4% for robotic, 92.5% for laparoscopic, and 90.9% for open reconstruction, with no statistically significant differences between techniques. However, the robotic approach was associated with a lower rate of major complications [7^▪^]. Another potential use of robotic or laparoscopic approach is the availability of technologies as indocyanine green fluorescence imaging, enabling real-time evaluation of ureteral perfusion and guiding resection margins to ensure graft placement on well-vascularized tissue [14^▪^].

## OUTCOMES AND DURABILITY

Definitions of success after oral mucosa graft ureteroplasty remain variable across series, but most authors judge success as no need for secondary intervention, no symptomatic obstruction, and stable or improved drainage and renal function on follow-up imaging [9^▪▪^,11^▪^].

### Success rate and follow-up

Chao et al. in the largest multi-institutional cohort to date, reported 92% freedom from additional intervention at a median 29-month follow-up in 163 patients, with a median time to failure at 10.2 months [9^▪▪^]. The authors concluded that post-operative surveillance should be intensified in the first year. Interestingly, in that cohort stricture length and prior treatment attempts did not emerge as independent predictors of failure, suggesting that patient selection and intraoperative judgement about tissue quality and graft bed may matter more than anatomy alone. Meta-analysis similarly supports high efficacy, with pooled success rates of 95% for buccal and 99% for lingual mucosa grafts across 436 patients [11^▪^]. In the most recent series, Zhou *et al.* reported favourable outcomes using lingual mucosa graft posteriorly augmented ureteroplasty, achieving 100% of success rates among 61 patients at 32 months of median follow up.

### Complications

Overall complication rates are reported in 18–28% of cases, mostly Clavien–Dindo grade II events such as urinary tract infection or minor wound complications [4^▪^,19^▪^]. The complication that deserves specific attention is urinary leak requiring percutaneous drainage (grade IIIa), which remains uncommon but is the early event most likely to prolong recovery and trigger downstream interventions.

### Donor-site morbidity

Oral graft harvest is generally well tolerated, and persistent donor-site morbidity appears uncommon in most contemporary series [12,19^▪^]. When symptoms occur, they are usually transient, including perioral numbness, discomfort, and short-lived limitation in mouth opening [11^▪^,^28^]. Lingual mucosa may offer a modest advantage in pooled analyses, but comparative data remain limited and standardized patient-reported outcome measures are rarely used [11^▪^]. In the last single-centre case series reported, Zhou *et al.* reported minimal donor-site morbidity, with oral symptoms limited to transient tongue discomfort or altered sensation that resolved spontaneously during follow-up, and no major donor-site complications requiring intervention [23].

## CONCLUSION

Oral mucosa graft ureteroplasty is now a credible, organ-preserving option for selected complex ureteral strictures, with most contemporary series reporting high rates of freedom from reintervention across different aetiologies and surgical approaches.

Contemporary multi-institutional series now report high rates of freedom from reintervention with low complication profiles [9^▪▪^,11^▪^].

Comparative syntheses suggest that successful outcomes are achievable across open, laparoscopic, and robotic approaches, underscoring that patient selection, technical execution, and adjunct vascularized coverage are the key drivers of durability rather than surgical access alone [7^▪^,^17^,19^▪^].

Recent work has further refined practical adjuncts, including perinephric or retroperitoneal tissue coverage as alternatives to omentum and, in carefully selected cases, reconstruction without omental wrapping altogether [12,23,24^▪^].

Most available data are retrospective, success definitions vary, and follow-up is often insufficient to make firm statements about long-term durability and long-term renal function evaluation.

Further studies are required, prospective, multicentre data collection using standardized endpoints, including patient-reported outcomes and longer-term renal function measures, to clarify selection criteria and identify the situations in which graft augmentation is most reliable.

## Acknowledgements

*None.*

### Financial support and sponsorship

*None.*

### Conflicts of interest

*There are no conflicts of interest.*

## REFERENCES AND RECOMMENDED READING

Papers of particular interest, published within the annual period of review, have been highlighted as:

▪ of special interest
▪▪ of outstanding interest
